# Elevated circulatory levels of leptin and resistin impair therapeutic efficacy of dacarbazine in melanoma under obese state

**DOI:** 10.1186/s40170-018-0176-5

**Published:** 2018-03-20

**Authors:** Parmanand Malvi, Balkrishna Chaube, Shivendra Vikram Singh, Naoshad Mohammad, Maleppillil Vavachan Vijayakumar, Snahlata Singh, Surbhi Chouhan, Manoj Kumar Bhat

**Affiliations:** National Centre for Cell Science, Savitribai Phule Pune University Campus, Ganeshkhind, Pune, 411 007 India

**Keywords:** Obesity, Leptin, Resistin, Melanoma, Chemotherapy, Weight-control interventions

## Abstract

**Background:**

Obesity is associated with increased risk, poor prognosis and outcome of therapy, in various cancers. Obesity-associated factors or adipokines, especially leptin and resistin, are purported to promote growth, survival, proliferation, and invasiveness of cancer cells. However, the mechanistic link between these adipokines and therapeutic response in malignancies is not clearly understood.

**Methods:**

ob/ob and db/db mouse models were used in this study to evaluate the role of leptin and resistin towards the outcome of dacarbazine (DTIC) therapy in melanoma. Unique in vitro approaches were employed to complement in vivo findings by culturing melanoma cells in the serum collected from the experimental mice.

**Results:**

Here, we have shown the role of important adipokines leptin and resistin in growth and the outcome of DTIC therapy in melanoma. Both leptin and resistin not only enhance proliferation of melanoma cells but also are involved in impairing the therapeutic efficacy of DTIC. Leptin and resistin treatment caused an increase in the protein levels of fatty acid synthase (FASN) and caveolin 1 (Cav-1) respectively, through their stabilization in A375 cells. Further, it was observed that leptin and resistin impaired the response of melanoma cells to DTIC via upregulation of heat shock protein 90 (Hsp90) and P-glycoprotein (P-gp) respectively.

**Conclusion:**

These findings unraveled the involvement of adipokines (leptin and resistin) in melanoma progression, and more importantly, in the outcome of DTIC therapy.

**Electronic supplementary material:**

The online version of this article (10.1186/s40170-018-0176-5) contains supplementary material, which is available to authorized users.

## Background

Obesity is characterized by increased accumulation of white adipose tissue in the body [1]. White adipose tissue secretes many cytokines and hormonal factors, which are collectively referred to as adipokines. In obese state, the secretion profile of adipose tissue is altered [2]. As a consequence of the alteration in secretion profile of adipose tissue, increased level of pro-inflammatory adipokines and a simultaneous decrease in anti-inflammatory adipokines is observed [3, 4]. Adipokines act through receptors or membrane-associated molecules, and mediate their effect by activating various cellular signaling pathways [5]. Altered levels of adipokines support cancer cells in terms of survival, growth, and proliferation, thereby promoting tumor development, angiogenesis, progression, and metastasis.

In addition to being an influential tumor-promoting factor, obesity has also been reported to have a critical impact on the outcome of therapeutic responses in certain cancers. Under the obese state, numerous key inflammatory and metabolic factors, and their pathways, are assumed to mediate the obesity-associated impairment of chemotherapeutic responses [6, 7]. The purported mechanisms underlying increased cancer risk in the obese state relate to multiple molecular and metabolic changes arising primarily as a consequence of adipose tissue expansion. The obesity-related changes include elevated levels of hormones and growth factors such as insulin, insulin-like growth factor (IGF)-1, and sex steroid hormones; adipokine imbalances; and a chronic state of low-grade inflammation. The altered systemic and local microenvironments that occur as a consequence of the obesity not only increase the likelihood of tumor development and progression but also potentially create an unfavorable state for response to chemotherapeutic regimens [7]. The mechanistic studies on the impact of the obese phenotype towards the outcome of cancer therapy are still lacking. There is a need to re-consider the dosing pattern of chemotherapeutic drugs with the concomitant implication of interventions, which curtail adiposity.

The involvement of adipose tissue in impairing the therapeutic response in cancers has been reported [8–10]. However, very little is known about the specific role of adipokines in the outcome of cancer therapy. Adipokines such as leptin and resistin are found to be elevated in the serum of obese individuals [11]. Leptin is known to activate various signaling pathways including PI3K/Akt, JAK/STAT, and MAPK. Its role in growth and proliferation has been extensively explored in breast and prostate cancers [12, 13]. However, the specific role of leptin in modulating melanoma cell proliferation and the chemotherapeutic outcome is obscure. Another important adipokine, resistin, is known to promote cancer growth. There is a considerable amount of experimental and epidemiological evidences which suggest that resistin may have pathophysiological effects, particularly in some cancer types, in addition to its traditional roles in energy homeostasis [14]. Moreover, resistin is also known to promote drug resistance phenotype in certain malignancies [15]. Previously, study from our group has shown that obesity impairs the therapeutic outcome of dacarbazine (DTIC) in melanoma and induces drug-resistant phenotype by upregulating fatty acid synthase (FASN), caveolin (Cav)-1, and P-glycoprotein (P-gp) [7]. In the present study, we investigated the specific role of leptin and resistin in melanoma cell growth, proliferation, and the outcome of DTIC-based chemotherapy. Using appropriate in vivo and in vitro approaches, we have shown that these adipokines not only modulate the growth and proliferation of melanoma cells but also are responsible for impairment in the efficacy of DTIC.

## Materials and methods

### Cell lines and culture conditions

Murine melanoma cells B16F10 and B16F1, human melanoma cells A375, and murine preadipocyte cells 3T3-L1 were procured from American Type Culture Collection (ATCC, Manassas, VA, USA). All the cell lines were maintained at our in-house cell repository at National Centre for Cell Science (NCCS), Pune, India. All the cells were routinely cultured in Dulbecco’s modified Eagles medium (Life Technologies, CA, USA) containing 25 mM glucose and supplemented with 10% heat-inactivated (56 °C for 30 min) fetal bovine serum (Hyclone, UT, USA, or Gibco, NY, USA), penicillin (100 U/ml), and streptomycin (100 μg/ml) (Invitrogen Life Technologies, CA, USA) and maintained at 37 °C in a 5% CO_2_ humidified incubator (Thermo Fisher Scientific, OH, USA).

### Chemicals and reagents

Dacarbazine, MCD, and geldanamycin (GA) were procured from Sigma (MO, USA), cerulenin was obtained from Calbiochem (CA, USA), and cycloheximide was purchased from ICN (CA, USA). Antibodies against Hsp90 (Rabbit polyclonal, sc-7947), TNF-α (Goat polyclonal, sc-1347), ob (leptin) (Rabbit polyclonal, sc-9014), and resistin (Goat polyclonal sc-16117) and HRP-conjugated secondary antibodies against rabbit, mouse, rat, and goat IgG were purchased from Santa Cruz Biotechnology, CA, USA. P-gp (Mouse monoclonal, cat.no. ab3364) antibody was purchased from Abcam, MA, USA. IL-6 (Rat monoclonal, cat no. 554400) antibody was purchased from BD Biosciences, NJ, USA. FITC and Rhodamine-conjugated secondary antibodies against mouse, rabbit, and goat IgG were purchased from KPL, Gaithersburg, Maryland, USA.

### Cycloheximide chase experiment

Approximately 3 × 10^5^ A375 cells were plated in 35-mm culture dishes. After 24 h, culture medium was replaced by DMEM medium supplemented with 1% FBS, and the cells were treated with leptin and resistin for 48 h. Next, cycloheximide (Chx) was added to the cells at a concentration of 100 μg/ml. Cells were further incubated for 0, 30, 60, and 120 min. Thereafter, cell lysates were prepared and subjected to Western blotting.

### Rhodamine-123 efflux assay

To measure efflux of Rhodamine-123 (Rh-123), reflective of transport activity for P-gp, melanoma cells were seeded at a density of 3 × 10^5^ cells in 35-mm culture dishes and allowed to adhere for 24 h. These cells were then grown in the presence or absence of leptin or resistin for 24 h. Cells were washed thrice with PBS and incubated for 30 min at 37 °C in PBS containing 2 μM Rh-123. The solution was removed and cells were detached from culture flask by incubating in trypsin-containing, Ca^2 + −^free, phosphate-buffered solution with 2 μM Rh-123. Trypsin digestion was stopped by adding cold PBS solution supplemented with 5% fetal bovine serum and 2 μM Rh-123. Cell aliquots were prepared in 1.5 ml tubes and centrifuged at 3000 rpm for 5 min at 4 °C. The supernatant was aspirated out, 2-ml cold incubation solution was added into the tubes and kept at 4 °C. The loading of Rh-123 at this point was considered as 0-min time point. Thereafter, the efflux of Rh-123 was initiated by incubating the cells at 25 °C for 30 min. Next, the fluorescence intensity of Rh-123 was measured through a 530/30 nm bandpass using FACS Calibur, and the data were analyzed using CellQuest Pro software (BD Biosciences, NJ, USA).

### MTT assay

Melanoma cells were plated at a density of 6 × 10^3^ cells/well in 96-well plates and allowed to adhere. After 24 h, cells were treated with vehicle (PBS or ethanol), drugs, or adipokines as per the experimental requirements. After required treatment time points, medium was removed and 50 μl of MTT (methyl thiazol tetrazolium, 1 mg/ml in DMEM without phenol red) (Sigma, MO, USA) was added to each well and further incubated for 4 h at 37 °C. MTT is reduced by mitochondrial dehydrogenase activity in metabolically active cells to form insoluble formazan crystals. Formazan crystals were solubilized in 100 μl of isopropanol and absorbance was measured at 570 nm using 630 nm as a reference filter (MultiSkan Go, Thermo Fisher Scientific, OH, USA). The absorbance given by untreated cells was taken as 100% cell survival.

### Long-term survival assay

B16F10, B16F1, and A375 cells were plated at an appropriate density of 1 × 10^3^ cells/well in 6-well plates. Next day, cells were treated with vehicle or drugs or inhibitors as per the experimental requirements. After 48 h, the medium was removed and fresh medium was added. Cells were allowed to grow for 10 days with medium change every 2–3 days. Thereafter, cells were fixed with 3% paraformaldehyde for 10 min and stained with 0.05% crystal violet for 2 h at room temperature. Images were taken using a digital camera (Olympus, Tokyo, Japan). Quantitation of colonies was performed by using NIH Image J software (Image J Freeware; http://rsb.info.nih.gov/ij/). The survival of control cells was considered as 100%.

### Experimental mice

Wild-type (WT)-ob, ob/ob, WT-db, and db/db mice (6–8 weeks aged, male or female) and C57BL/6J mice were used in the present study. WT-ob, ob/ob, WT-db, and db/db mice were procured from Jackson Laboratories (ME, USA) and were maintained in Experimental Animal Facility (EAF) at National Centre for Cell Science (NCCS), Pune, India. Mice were housed and maintained in animal quarters under environmentally controlled conditions (22 ± 2 °C) with a 12-h light/dark cycle and had free access to water and standard rodent pellet food (Golden feeds, New Delhi, India) *ad libitum*, unless otherwise stated. For developing diet-induced obesity, C57BL/6J mice were fed with high fat diet (HFD) as described previously [7]. All animal experiments were carried out as per the requirement and guidelines of the Committee for the Purpose of Control and Supervision of Experiments on Animals (CPCSEA), Government of India, and after obtaining permission of the Institutional Animal Ethics Committee (IAEC).

### Calorie restriction (CR) in ob/ob and db/db mice, tumor challenge, DTIC administration, and follow-up

To study the impact of leptin on the outcome of DTIC therapy in melanoma, B16F10 melanoma isograft was induced in ob/ob and db/db mice by injecting cells (2 × 10^5^) in 100 μl of PBS subcutaneously (sc). After the appearance of the palpable tumor, mice were divided into four groups. Group 1 mice were kept as control, group 2 mice were treated with DTIC, group 3 mice were kept on 50% caloric restrictions (CR), and group 4 mice were kept on CR followed by DTIC treatment (80 mg/kg intraperitoneally for 5 consecutive days as described previously) [7]. The detailed experimental plan is illustrated in Fig. 4a. Tumor volume was calculated using the formula: 0.52 × length × width^2^ and was followed up throughout the study. At the end of the experiment, mice were sacrificed by CO_2_ euthanasia. Excised tumors volume and weight were recorded, and the samples were immediately preserved at − 80 °C until further use. To observe their survival rates, five mice from each group were followed up for an additional 60 days.

### Serum biochemical analysis

Glucose level was measured using rapid glucose analyzer (Accu-Chek Sensor Comfort, Roche Diagnostics, Mannheim, Germany) by collecting blood through an approved tail cap method. For serum collection, blood was collected by orbital sinus puncture and centrifuged at 6000 rpm at room temperature. Triglycerides (TG), cholesterol, LDLc, and free fatty acids levels in fresh serum were estimated using colorimetric kits (Spinreact, Girona, Spain) as per the manufacturer’s instructions. Insulin, leptin, and adiponectin levels in the serum were estimated by mouse-specific respective ELISA kits as per the manufacturer’s protocol. Leptin, resistin, IL-6, and TNF-α levels in the serum were detected by indirect ELISA. Briefly, a standard curve was prepared with different concentrations of respective recombinant proteins. ELISA plates (Becton Dickenson, NJ, USA) were coated with serum samples collected from mice. Blocking was done using 2% BSA in phosphate buffered saline (PBS; pH = 7.4). After washing with PBS, samples were incubated with primary antibodies (1:100) for resistin, IL-6, and TNF-α specified for ELISA. Following washing, samples were incubated with HRP-conjugated secondary antibodies (1:200). ABTS [2,2′-azinobis-(3-ethylbenzothiazoline-6-sulfonic acid)] (Sigma, MO, USA) was used as a chromogenic substrate for HRP. After developing the color, absorbance was recorded at 405 nm.

### Reverse transcription PCR (RT-PCR)

Total RNA from treated and untreated cells was extracted using TRIzol™ reagent (Invitrogen, Carlsbad, USA), according to the manufacturer’s instructions. Cells (5 × 10^5^) were seeded in 35-mm plates and allowed to adhere for 24 h. Treatment conditions are indicated in respective experiments. Cells were directly lysed in culture plate by adding 1 ml of TRIzol reagent, and passing the cell lysates several times through the pipette tip, and collected in a 1.5-ml tube. Homogenized samples were incubated at room temperature for 5 min, and 200 μl chloroform was added. The contents in tubes were mixed by gentle shaking and incubated for 2–3 min at room temperature. Next, the tubes were centrifuged at 12,000×g for 15 min at 4 °C. Following centrifugation, the upper colorless aqueous phase was collected in fresh tubes. RNA was precipitated from the aqueous phase by addition of 500 μl isopropanol. Tubes were incubated for 10 min at room temperature and centrifuged at 12,000×*g* for 10 min at 4 °C. Supernatant was removed, and RNA pellet was washed once with 1 ml of 75% ethanol in DEPC-treated water by mixing and centrifuging at 7500×*g* for 5 min at 4 °C. At the end, RNA pellets were briefly air dried and dissolved in DEPC-treated water at 55 °C for 10 min.

### Culture of melanoma cells in serum collected from experimental ob/ob and db/db mice

Serum collected from experimental ob/ob, db/db, and their WT counterparts was pooled from respective groups. Approximately 1.5 × 10^2^ B16F10 cells were plated in 24-well plates and allowed to adhere. After 24 h, DMEM containing 5% serum collected from experimental mice was added and cells were cultured chronically for 10 days. The medium was changed on every 2–3 days. Finally, cells were fixed with paraformaldehyde, stained with crystal violet, and images were taken (as described above).

### Treatment with adipokines in vitro

To study the effect of leptin and resistin, recombinant human leptin and resistin (Sigma, MO, USA) were used to treat melanoma cells in vitro. A375 cells were plated in culture dishes or 6-well plates in DMEM containing 10% FBS. After 24 h, the medium was removed and cells were treated with varying concentrations (range 0.01–100 ng/ml) of leptin and resistin in DMEM containing 1% FBS for 24 or 48 h as per the experimental requirements. Treated cells were then analyzed by MTT assay or processed for immunoblotting or RT-PCR or confocal staining.

### Immunodepletion of leptin and resistin from serum collected from mice

Serum from HFD C57BL/6 J mice was collected, and pooled (as described above). Leptin and resistin (or both together) were immunodepleted from the serum by incubating it with respective specific antibody (Santa Cruz Biotechnology, CA, USA), at 4 °C for overnight. Antigen-antibody complexes were precipitated using protein A/G-plus agarose beads (Santa Cruz Biotechnology, CA, USA) by incubating at 4 °C for 4 h. Next, the supernatant containing immunodepleted serum was collected by centrifuging the tubes at 10,000 rpm at 4 °C. Following validation of immunodepletion of leptin and resistin in the serum (Additional file 1: Figure S1A and S1B), B16F10 or B16F1 cells (3 × 10^5^) seeded in 35-mm dishes were cultured in DMEM containing 5% immunodepleted serum. After 48 h, the cells were harvested and lysates were prepared for immunoblotting.

### Statistical analysis

Statistical analysis was performed using Sigma Plot 12.0 (Systat Software Inc., CA, USA). All data were represented as the mean ± standard error of the mean (S.E.M.). All in vitro experiments were performed at least three times unless otherwise mentioned. For in vivo experiments involving more than two groups, one-way ANOVA was used, followed by the Tukey multiple comparison test. In vitro or in vivo data involving two experimental groups were analyzed using two-tailed unpaired Student’s *t* test. The values of *p* < 0.05, *p* < 0.01, and *p* < 0.001 were considered as statistically significant (*), very significant (**), and highly significant difference (***) respectively, unless otherwise mentioned.

## Results

### Leptin and resistin impair the efficacy of DTIC in melanoma cells

**Fig. 1:**
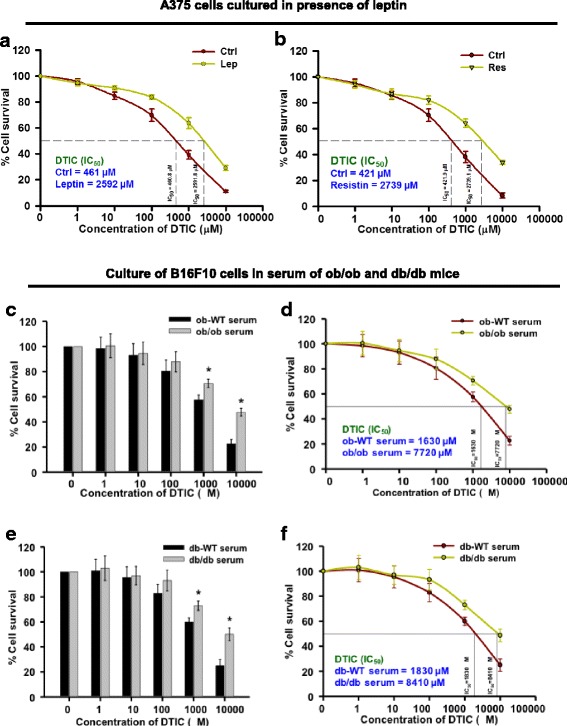
Leptin and resistin impair the outcome of DTIC therapy in melanoma cells. **a**, **b** Effect of leptin (**a**) and resistin (**b**) on the outcome of DTIC in A375 cells. A375 (human melanoma) cells were plated in 96-well plates. After 24 h, cells were treated with 100 ng/ml of recombinant leptin or resistin in DMEM containing 1% FBS for 1 h. Then, cells were treated with DTIC at the indicated concentrations and incubated for 48 h. These cells were subjected to MTT assay, and IC_50_ of DTIC was calculated. **c**–**f** Effect of ob/ob and db/db serum factors on the survival of B16F10 cells upon DTIC treatment. B16F10 cells were chronically grown in medium containing 5% serum collected from ob-WT or ob/ob mice for 15 days. These cells were then subjected to DTIC treatment at the indicated concentrations, for 48 h, and MTT assay was performed. **c** Survival of B16F10 cells in DMEM containing ob-WT or ob/ob serum. **d** Measurement of IC_50_ values of DTIC in DMEM containing ob-WT or ob/ob serum. **e**, **f** B16F10 cells were chronically grown in medium containing 5% serum collected from db-WT or db/db mice for 15 days. These cells were then subjected to DTIC treatment at the indicated concentrations, for 48 h, and MTT assay was performed. **e** Survival of B16F10 cells in DMEM containing db-WT or db/db serum. **f** Measurement of IC_50_ value of DTIC in DMEM containing db-WT or db/db serum. The data are representative of experiments performed three times in triplicate. The results are given as means ± standard error of the mean. All the experiments were performed three times. Statistical analysis was performed using two-tailed unpaired Student’s *t* test; *p < 0.05; Ctrl, control; Lep, leptin; Res, resistin

Previously, we have reported that obesity impairs efficacy of DTIC in melanoma, which is mediated by FASN and Cav-1 [7]. To unravel the mechanism as to how obesity impairs the outcome of DTIC therapy in melanoma, we investigated the specific role of leptin and resistin as their levels in serum are elevated under obese condition. Firstly, to explore whether leptin and resistin could have any role in modulating the sensitivity of melanoma cells to DTIC, A375 cells were treated with DTIC in the presence or absence of leptin or resistin. We observed that leptin significantly impaired the response of A375 cells to DTIC as evident from the increase in IC_50_ of DTIC by ~ 5-fold (2592 μM) as compared to the control (461 μM) (Fig. 1a). Similarly, in A375 cells treated with resistin IC_50_ for DTIC increased to 2739 μM as compared to control (421 μM) (Fig. 1b).

Additionally, to verify the involvement of leptin in modulating the response of melanoma cells to DTIC therapy, B16F10 cells were cultured in the medium containing serum of genetically obese mice strains ob/ob (leptin deficient) or db/db (leptin receptor deficient). As compared to ob-WT serum, the cytotoxicity of DTIC was found to be impaired in ob/ob serum (Fig. 1c). Interestingly, the IC_50_ value of DTIC was increased (7720 μM) in cells grown in ob/ob serum as compared to those cultured in ob-WT serum (1630 μM) (Fig. 1d). Similarly, the response of melanoma cells to DTIC was found to be reduced in the presence of ob/ob serum when compared to that of db-WT serum (Fig. 1e). Higher IC_50_ value of DTIC was observed in melanoma cells grown in the serum of db/db mice (8410 μM), which contains very high circulatory leptin, as compared to control (1830 μM) (Fig. 1f).

### Diminished circulatory level of leptin and resistin improves the efficacy of DTIC in melanoma cells

**Fig. 2:**
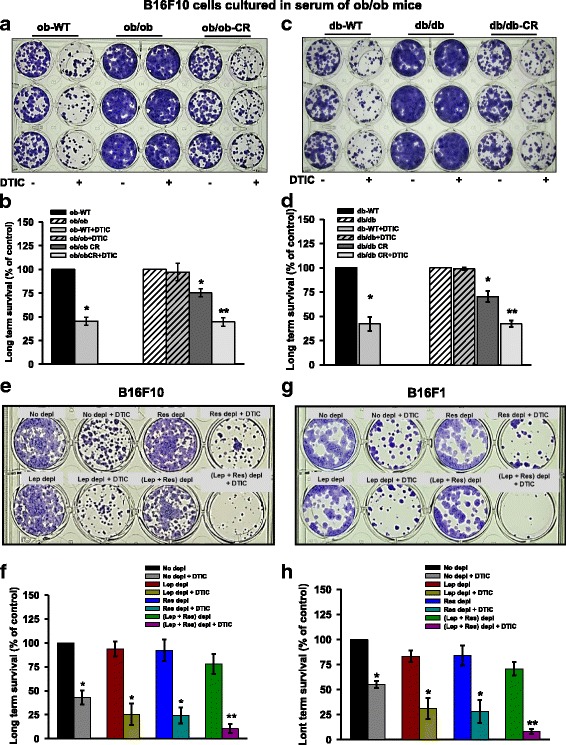
Effect of obesity-associated serum factors on the long-term survival of B16F10 and B16F1 cells treated with DTIC. **a**, **b** B16F10 cells were chronically grown in the medium containing 5% serum collected from experimental ob/ob mice for 15 days. Thereafter, these cells were subjected to DTIC treatment for 48 h. Then, the medium was changed and fresh medium was added. The medium was changed every 2–3 days. **a** Representative image showing the long-term survival of B16F10 cells. **b** Bar graph showing the quantitation of number of surviving population from the image shown in **a**. **c**, **d** Similar experiment was performed in B16F10 cells chronically grown in the serum from db/db mice. After 10 days, the cells were stained with 0.05% crystal violet, and images were taken using Olympus digital camera. **e**–**h** B16F10 and B16F1 cells were cultured in serum (collected from C57BL/6J mice) which was immuno-depleted of leptin and or resistin for 48 h. Then, DTIC treatment was given, and cells were incubated for 48 h. Next, the medium was changed and fresh medium was added. **e** Representative image showing the long-term survival of B16F10 cells. **f** Bar graph showing the quantitation of number of surviving population from the image shown in **e**. **g** Representative image showing the long-term survival of B16F1 cells. **h** Bar graph showing the quantitation of number of surviving population from **g**. The data are representative of experiments performed two times at least in triplicates. The data were quantified using Image J software. The results are given as means ± standard error of the mean. Statistical analysis was performed using one-way ANOVA, followed by the Tukey multiple comparison test (for **b** and **d**), whereas two-tailed unpaired Student’s *t* test was used (for **f** and **g**). *p < 0.05, **p < 0.001; Ctrl, control; Lep, leptin; Res resistin

Further, to complement the role of leptin and resistin and other obesity-associated factors in cell growth, long-term cell survival assay was performed. Melanoma cells were cultured in the medium containing serum from ob/ob or db/db mice or the caloric restricted (CR), in the presence or absence of DTIC. Enhanced cell growth and proliferation was observed in B16F10 cells grown in the medium containing the serum from ob/ob or db/db mice as compared to those cultured in the medium containing serum from their WT counterparts in the long-term culture. Importantly, in the long-term culture, we observed impairment in the efficacy of DTIC in the B16F10 cells grown in the medium containing the serum from ob/ob (Fig. 2a, b) or db/db (Fig. 2c, d) mice as compared to the control. Moreover, the effect of DTIC was markedly rescued upon culturing these cells in the medium containing serum of calorically restricted ob/ob (Fig. 2a, b) or db/db mice (Fig. 2c, d).

To further check whether modulation of chemotherapeutic outcome by obesity-associated factors is dependent on leptin or resistin, B16F10 and B16F1 cells were cultured in the medium containing HFD-C57BL/6J serum which was immuno-depleted of leptin or resistin by respective antibodies either alone or together. Interestingly, we observed that leptin depletion significantly improved the efficacy of DTIC as evident by decrease in the number of colonies in long-term survival assay as compared to the cells grown in the medium containing the control serum [(B16F10: Fig. 2e, f) and (B16F1: Fig. 2g, h)]. Similarly, resistin depletion resulted in improved efficacy of DTIC in melanoma cells as compared to the cells grown in the medium containing the control serum [(B16F10: Fig. 2e, f) and (B16F1: Fig. 2g, h)]. Interestingly, upon simultaneous immuno-depletion of both leptin and resistin, the effect of DTIC was prominently improved [(B16F10: Fig. 2e, f) and (B16F1: Fig. 2g, h)].

### Leptin- and resistin-induced impaired response of melanoma cells to DTIC is mediated by FASN/Hsp90 and Cav-1/P-gp respectively

**Fig. 3:**
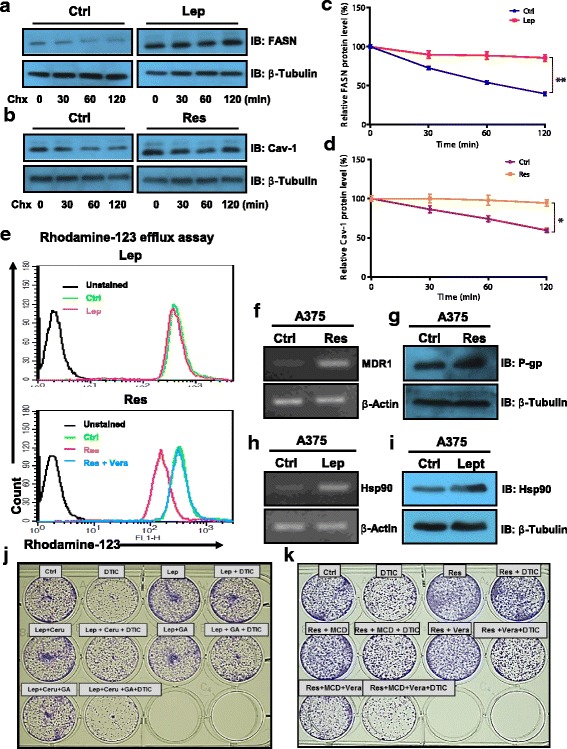
Molecular events associated with leptin and resistin induced impaired outcome of DTIC therapy in melanoma cells. **a**, **b** A375 cells were treated with leptin or resistin at a concentration of 100 ng/ml in DMEM containing 1% FBS for 48 h as described in the “Materials and methods” section. Thereafter, cycloheximide (100 μg/ml) treatment was given for the indicated time points. Representative immunoblot of FASN (**a**) and Cav-1 (**b**) in A375 cells treated with leptin or resistin respectively. **c**, **d** Bar graph showing the quantitation of band intensity of FASN and Cav-1 immunoblots. **e** Rhodamine-123 efflux assay in A375 cells treated with leptin (upper panel) or resistin (lower panel). A375 (human melanoma) cells were plated in 12-well plates. After 24 h, cells were treated with 100 ng/ml of recombinant leptin in DMEM containing 1% FBS for 48 h. Thereafter, these cells were subjected to Rh-123 efflux assay via flow cytometry. **f**, **g** RT-PCR (**f**) and immunoblotting (**g**) analysis of MDR and P-gp respectively in A375 cells treated with resistin. **h**, **i** RT-PCR (**h**) and immunoblotting (**i**) analysis of HSP90 in A375 cells treated with leptin. **j**, **k** Representative image showing the long-term survival of A375 cells grown in the presence or absence of leptin (**j**) or resistin (**k**) together with inhibitors. The results are given as means ± standard error of the mean. All the experiments were performed three times. Statistical analysis was performed using two-tailed unpaired Student’s *t* test for **c** and **d**. *p < 0.05, **p < 0.001; Ctrl, control; Lep, leptin; Res, resistin; Chx, cycloheximide; Ceru or C, cerulenin; GA or G, geldanamycin

To get insights into the role of leptin and resistin in causing attenuation in the response to DTIC, we intended to analyze the status of FASN and Cav-1 which are upregulated in melanoma in the obese (HFD) mice [16], and are known to be involved in resistance to cancer chemotherapy [17]. Firstly, we checked the protein levels of FASN and Cav-1 in A375 cells upon treatment with leptin or resistin. An increased level of FASN was detected in A375 cells upon leptin treatment (Fig. 3a), while resistin treatment caused increase in the level of Cav-1 in A375 cells (Fig. 3b). Previously, it has been reported that leptin and resistin do not affect the mRNA expression of FASN and Cav-1 at transcription levels; however, they increase protein level of these molecules [18]. Therefore, these findings suggested that both leptin and resistin modulate the protein levels of FASN and Cav-1 respectively, likely by increasing the stability of these proteins. To verify this, cycloheximide chase experiment was performed in A375 cells. We observed that leptin promoted stabilization of FASN, whereas resistin caused Cav-1 stabilization (Fig. 3c, d, respectively) in these cells.

Another important molecule primarily responsible for pumping out the anticancer drugs from cancer cells thereby rendering them resistant to chemotherapy is P-gp [19]. To confirm the involvement of P-gp in decreasing the response to DTIC in the presence of leptin or resistin, Rh-123 efflux assay was performed. We noticed that leptin did not affect Rh-123 efflux in A375 cells (Fig. 3e, upper panel). On the other hand, resistin did increase the efflux of Rh-123 in A375 cells, which was reversed upon treatment of verapamil, an inhibitor of P-gp (Fig. 3e, lower panel). Moreover, it was observed that resistin increased P-gp mRNA as well as protein levels (Fig. 3f, g), suggesting that resistin plays a role in chemotherapeutic outcome in melanoma, in part, via increasing the expression of P-gp, while leptin-mediated impaired response of cancer cells to DTIC is independent of P-gp activity. This led us to explore another possible mechanism by which leptin contributes to the impaired DTIC action on melanoma cells. It has been reported that leptin modulates the levels of heat shock proteins (Hsps) [20, 21]. Moreover, Hsp90 is one of the major heat shock proteins known to contribute to drug-resistant phenotype [22, 23]. Therefore, we analyzed the involvement of Hsp90 and checked the expression of Hsp90 in melanoma cells upon treatment with leptin. We found that leptin treatment indeed increased transcript and protein levels of Hsp90 in A375 cells (Fig. 3h, i).

To confirm whether FASN and Hsp90 are involved in leptin-induced impairment in the response of melanoma cells to DTIC, we used their respective inhibitors. Inhibition of FASN and Hsp90 individually by cerulenin and geldanamycin respectively increased the sensitivity of A375 cells to DTIC even in the presence of leptin (Fig. 3j). Moreover, combined inhibition of FASN and Hsp90 enhanced the effect of DTIC in A375 cells compared to single inhibitor treatment (Fig. 3j; Additional file 1: Figure S2A). Similarly, to verify the role of Cav-1 and P-gp in impairing the response to DTIC by resistin, their inhibitors MCD and verapamil respectively were used. Inhibition of these molecules resulted in increased sensitivity of A375 cells to DTIC (Fig. 3k). Simultaneous inhibition of both of these molecules profoundly improved the anticancer effect of DTIC (Fig. 3k; Additional file 1: Figure S2B).

### Elevated serum level of leptin and resistin is correlated with enhanced melanoma growth and impaired efficacy of DTIC in vivo

To corroborate the involvement of leptin in melanoma growth and in the outcome of DTIC treatment, we employed ob/ob and db/db mice. Leptin is an important adipocyte-secreted factor involved in controlling appetite. Due to lack of functional leptin and leptin receptor respectively, ob/ob and db/db mice are morbidly obese (despite fed on a normal fat diet) exhibiting higher level of fat accumulation. Thus, these mice serve as good models for the leptin-related studies.

**Fig. 4:**
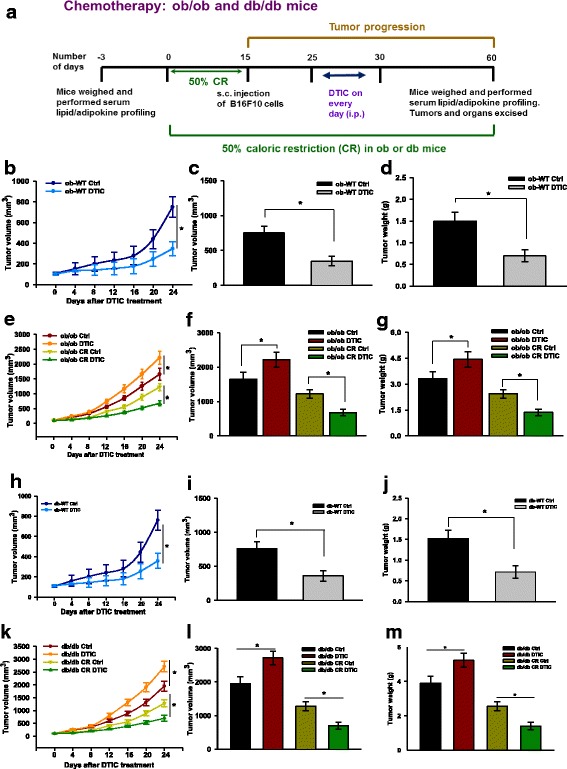
Impact of leptin on melanoma progression and on the outcome of DTIC therapy in ob/ob and db/db mice. **a** Layout of the in vivo experiments. **b**–**d** ob-WT mice were injected with B16F10 cells (2 × 10^5^ cells/mouse in 100 μl PBS). After the tumor formation, vehicle or DTIC treatment (*N* = 6 per each group) was given as per the experimental layout shown in **a**. **b** Tumor progression, **c** tumor volume, and **d** tumor weight. **e**–**g** ob/ob mice were divided into two major groups. One group was fed *ad libitum* on normal diet. In the second group, caloric intake was restricted to 50% by providing half the quantity of feed in normal before inoculating B16F10 cells. After 15 days, mice of all groups were injected subcutaneously with B16F10 cells (2 × 10^5^ cells/mouse in 100 μl PBS). After tumor formation, vehicle or DTIC treatment (*N* = 6 per each group) was given as per the experimental layout shown in **a**. **e** Trend of tumor progression, **f** tumor volume, and **g** tumor weight. **h**–**j** db-WT mice were injected with B16F10 cells (2 × 10^5^ cells/mouse in 100 μl PBS). After tumor formation, vehicle or DTIC treatment (*N* = 6 per each group) was given as per the experimental layout shown in **a**. **h** Trend of tumor progression, **i** tumor volume, and **j** tumor weight. **k**–**m** db/db mice were divided into two major groups. One group was fed *ad libitum* on normal diet. In the second group, caloric intake was restricted to 50% by providing half the quantity of feed in normal before inoculating B16F10 cells. After 15 days, mice of all groups were injected subcutaneously with B16F10 cells (2 × 10^5^ cells/mouse in 100 μl PBS). After tumor formation, vehicle or DTIC treatment (*N* = 6 per each group) was given as per the experimental layout shown in **a**. At the end of the experiment, mice were sacrificed and tumors were collected. **k** Trend of tumor progression, **l** tumor volume, and **m** tumor weight. The results are given as means ± standard error of the mean. Statistical analysis was performed using two-tailed unpaired Student’s *t* test (**b**, **h**), whereas one-way ANOVA, followed by the Tukey multiple comparison test was used for **e** and **k**. *p < 0.05, when compared to respective controls

To understand the involvement of leptin in melanoma progression, and the outcome of chemotherapy, ob/ob and db/db mice, as well as their WT counterparts, were ectopically isografted with B16F10 cells as shown in Fig. 4a. After the appearance of palpable tumors, DTIC was administered for 5 consecutive days and tumor progression was followed up until the termination of the experiment. We observed that DTIC treatment did not affect the serum levels of obesity-associated factors in ob/ob (Additional file 1: Table S1) and db/db mice (Additional file 1: Table S2) as well as in their WT littermates. As expected, DTIC significantly retarded tumor progression in respective WT counterparts, as evident by reduced tumor volume and weight (Fig. 4b–d, h–j). Surprisingly, therapeutic efficacy of DTIC was impaired in both ob/ob and db/db mice as is evident by the increase in tumor progression as compared to respective untreated controls (Fig. 4e–g, k–m).

In previous study, we have shown that shifting mice from HFD to ND has a profound impact on melanoma progression, which correlates with normalization in the body weight and in the levels of obesity-associated factors [18]. Therefore, to determine whether caloric restriction (CR) could improve the effect of DTIC in ob/ob and db/db mice, we subjected these mice to CR by reducing their feed by 50% as shown in Fig. 4a. In the present study, it was observed that CR itself reduced tumor progression in both ob/ob and db/db mice which correlated with the limited normalization in the levels of obesity-associated factors (Additional file 1: Table S1 and Table S2). More importantly, CR significantly improved the outcome of DTIC therapy in these mice (Fig. 4e–g, k–m).

## Discussion

Obesity is associated with an elevation in the levels of many pro-inflammatory and proliferative factors, which contribute to increased growth and proliferation of cancer cells [24]. Furthermore, in vitro studies have revealed growth-promoting effect of serum from ob/ob mice on cultured melanoma cells [25]. Pro-inflammatory (such as IL-6 and TNF-α) and proliferative (like leptin and insulin) factors are considered as tumor growth favoring molecules [26–28]. Both leptin and resistin are two of the important adipokines, which are elevated in obesity. Although the involvement of leptin and resistin in malignancies such as breast and prostate cancers has been extensively studied, very little is known about their role in melanoma growth and chemotherapeutic outcome. For better understanding and management of obesity-promoted malignancies, the involvement of obesity-associated factors needs to be comprehensively investigated [29]. The present study is aimed at unraveling the specific role of leptin and resistin in melanoma growth and the chemotherapeutic outcome using appropriate in vitro and in vivo approaches.

As observed in our previous study, by controlling obesity, tumor growth is restricted partly through normalization in the serum levels of obesity-associated factors such as leptin and resistin [18]. It has been shown that leptin and resistin stimulate the Akt signaling in cancer cells [30, 31], and hyperactivated Akt pathway was associated with increased protein levels of FASN and Cav-1 [16]. Our in vitro studies show that treatment of melanoma cells with leptin or resistin leads to increased levels or activation of proteins which are associated with regulation of cellular growth and metabolism, indicating their involvement in promoting melanoma cell growth and proliferation, which is consistent with findings of other research groups [25, 32]. Previously, we have examined the effect of various concentrations of both leptin and resistin on cell survival [18]. As the data indicated in the study, the optimum dose response was found to be 100 ng/ml, which was used throughout the study. Owing to the limitations in replicating the physiological conditions precisely in cell based experiments, especially, when the availability and dynamics/kinetics of these cytokines vary in vitro and in vivo, it would be inappropriate to compare the level of these adipokines present in vivo with the concentration used in vitro. The levels of cytokines are, generally, maintained at steady level in vivo. On the other hand, considering the half-life and artificially maintained physiology, higher concentrations of adipokines are required under in vitro conditions. Therefore, to sustain the effect of these adipokines during the course of in vitro studies, we relied on higher concentrations. Also, there are number of studies which used similar concentration of these adipokines for other related studies [33–36].

From a molecular point of view, leptin and resistin specifically enhanced FASN and Cav-1 protein level respectively. Intriguingly, there was no change in the transcript level of FASN or Cav-1 in cells treated with leptin or resistin. However, cycloheximide chase experiment confirms that leptin and resistin cause the stabilization of FASN and Cav-1 protein levels respectively in melanoma cells. Considering our previous study on HFD mice [7], normalized serum levels of these adipokines, via reduced adiposity, could be associated with a reduction in FASN and Cav-1 protein levels and decrease in Akt activation, leading to reduced progression of melanoma in the experimental HFD mice.

Fatty acid metabolism plays a pivotal role in various aspects of cancer progression. It has been recently shown that adipocyte-derived fatty acids drive breast cancer cell proliferation and migration by promoting adipocyte lipolysis [37]. In the present study, we observed that leptin and resistin cause impairment in the effect of DTIC on melanoma cells in vitro. In addition to tumor promoting effect on cancer cells [38–41], FASN and Cav-1 have been reported to protect cancer cells from chemotherapy by inducing drug resistance [17]. Since FASN and Cav-1 were modulated by leptin and resistin respectively, the impairment in chemotherapeutic outcome of DTIC could be partly due to the involvement of FASN and Cav-1 proteins. Another protein with critical role in tumor growth and cancer drug resistance is Hsp90 [42, 43]. Hsp90 is also involved in the stabilization of various molecules required for tumor growth [44–47]. In this study, we show that leptin, in addition to stabilizing FASN protein, enhanced the protein and mRNA level of Hsp90. Thus, the mutual involvement of FASN and Hsp90 could contribute to the unresponsiveness of melanoma cells to DTIC in the presence of leptin. To confirm this proposition, we show that simultaneous inhibition of FASN and Hsp90 together, in the leptin-treated A375 cells, indeed immensely enhanced the cytotoxic effect of DTIC. Another mechanism that stimulates drug-resistant phenotype is overexpression of P-gp in order to prevent cytotoxic effects of anticancer drugs [48]. In this study, resistin was found to increase P-gp mRNA and protein levels. Therefore, resistin-mediated impairment of DTIC treatment in melanoma cells could be due to collective involvement of Cav-1 and P-gp, both being integral component of plasma membrane of cancer cells. Similar to FASN and Hsp90 inhibition together in leptin-stimulated cells, simultaneous inhibition of Cav-1 and P-gp in resistin-treated A375 cells prominently reduced survival upon exposure to DTIC. Consistent with these findings, improvement in the effect of DTIC was observed upon culturing B16F10 and B16F1 cells in mice serum immuno-depleted of leptin and resistin.

As observed in our in vivo studies, irrespective of the presence of leptin, we noticed the impairment of DTIC therapy in ob/ob and db/db mice. Nevertheless, our in vitro data clearly demonstrate the growth promoting and chemotherapy-impairment effect of leptin. This implies that leptin, in part, likely plays an important role in tumor growth as well as in the outcome of DTIC therapy. In addition, since we observed a trend of tumor progression and impaired therapeutic outcome in ob/ob mice (which lack functional leptin) as compared to their wild-type counterparts, obesity-associated factors (other than leptin) could also influence tumor progression and the impairment of chemotherapy. As serum level of resistin is very high in ob/ob mice, and we observed in vitro that resistin is equally partly responsible for causing impairment in the efficacy of DTIC, it is conceivable that even in the absence of leptin, resistin could be involved in impairing the efficacy of DTIC in ob/ob mice, as resistin is very well known to induce drug-resistant phenotype in various cancers [14, 15, 49, 50]. Although, our in vitro results established the fact that both leptin and resistin are involved in impairing the response of melanoma cells to DTIC, it is difficult to validate their role in vivo due to lack of resistin knockout animals as well as resistin neutralizing antibody. Moreover, CR in both ob/ob and db/db mice significantly reduced tumor growth and improved the efficacy of DTIC which correlated with normalization of the serum levels of obesity-associated factors. As per observations from our laboratory and other research groups [51–53], ob/ob and db/db mice consume ~ 6 g diet per day, while their WT counterpart mice eat ~ 3 g diet per day (which is essentially 50% of the diet consumed by ob/ob and db/db mice). Therefore, to mimic the diet consumption similar to their WT counterparts, ob/ob and db/db mice were restricted to 50% food intake. The culture of B16F10 and B16F1 cells in the presence of serum from the experimental ob/ob and db/db mice confirms that obesity-associated circulating factors are important for melanoma progression. Culturing melanoma cells in medium containing serum of calorically restricted (CR) ob/ob and db/db mice reveals that normalization in the levels of obesity-associated factors has a profound impact on tumor growth and outcome of chemotherapy. Thus, obesity-associated factors including leptin and resistin are crucial for cancer cell growth as well as for outcome of therapeutic response. However, the possibility of the involvement of other obesity-associated factors (including adipokines and nutritional factors) in cancer progression and chemotherapeutic outcome cannot be ruled out. Our results clearly indicated that leptin and resistin partly contribute to melanoma progression and in impairing the outcome of dacarbazine therapy. Host factors other than leptin and resistin (including insulin, other adipokines and nutritional factors) could also play a pivotal role. Nonetheless, our in vitro studies (especially treatment of melanoma cells with leptin and resistin, as well as their immune-depletion) demonstrate that these adipokines critically affect melanoma growth, and the chemotherapeutic outcome, by modulating melanoma promoting proteins. Moreover, our data show that depletion of leptin and resistin from the serum collected from obese mice resulted in the improved response of melanoma cells as compared to the undepleted serum suggestive of their role in modulating the response of melanoma cells to DTIC. Currently, our laboratory is actively pursuing to understand the role of other obesity-associated factors (including both adipokines and nutritional factors) on cancer progression. Collectively, these data suggest that both leptin and resistin partly play a crucial role in melanoma growth and adversely affect the chemotherapeutic outcome by modulating the molecules which are involved in tumor growth and drug resistance. In a nutshell, this study highlights the role of leptin and resistin in melanoma growth, and impairment in the chemotherapeutic outcome.

## Conclusions

**Fig. 5:**
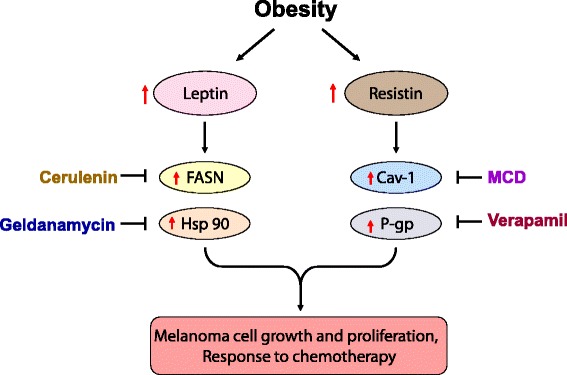
Proposed model of study on impact of leptin on melanoma growth and the outcome of dacarbazine therapy. Leptin modulates FASN and Hsp90 levels while resistin modulates Cav-1 and P-gp, thereby enhancing melanoma growth. Collectively, these events are responsible in part for impaired outcome of DTIC therapy. Inhibition of these molecules restricts melanoma growth and improves the outcome of chemotherapy

To conclude, this study investigates the role of increased levels of obesity-associated important adipokines leptin and resistin in melanoma growth and the outcome of DTIC-based therapy. Both of these adipokines activate Akt in melanoma cells. Leptin modulates FASN and Hsp90, while resistin increases Cav-1 and P-gp levels, thereby enhancing melanoma growth, and impairing the chemotherapeutic outcome (see the schematic overview in Fig. 5). Thus, this study provides a mechanistic link between these two adipokines and melanoma progression, as well as outcome of chemotherapy. Overall, our study suggests that controlling the circulating levels of these adipokines by life style interventions or inhibition of their molecular effectors is critical for better management of obesity-promoted cancer progression, and in improving the outcome of chemotherapy.

## Additional file

Additional file 1: **Figure S1.** Validation of immunodepletion of leptin form serum collected from HFD C57BL/6J mice. **Figure S2.** A375 cells were cultured in the presence of leptin or resistin along with inhibitors for 48 h. **Table S1.** Evaluation of obesity-associated factors in WT and db/db mice. **Table S2.** Evaluation of obesity-associated factors in WT and db/db mice. (DOC 319 kb)
