# Supplementary material for: Elevated circulatory levels of leptin and resistin impair therapeutic efficacy of dacarbazine in melanoma under obese state
Source: Cancer Metab. 2018 Mar 20;6:2. doi: 10.1186/s40170-018-0176-5 (PMC5859707; doi:10.1186/s40170-018-0176-5)
Supplement: Supplementary file 1 — Figure S1. Validation of immunodepletion of leptin form serum collected from HFD C57BL/6J mice. Figure S2. A375 cells were cultured in the presence of leptin or resistin along with inhibitors for 48 h. Table S1. Evaluation of obesity-associated factors in WT and db/db mice. Table S2. Evaluation of obesity-associated factors in WT and db/db mice. (DOC 319 kb) [file 40170_2018_176_MOESM1_ESM.doc]

**Title: Elevated circulatory levels of leptin and resistin impair therapeutic efficacy of dacarbazine in melanoma under obese state**

**Authors:** Parmanand Malvi, Balkrishna Chaube, Shivendra Vikram Singh, Naoshad Mohammad, Maleppillil Vavachan Vijayakumar, Snahlata Singh, Surbhi Chouhan and Manoj Kumar Bhat^,*^

National Centre for Cell Science, Savitribai Phule Pune University Campus, Ganeshkhind, Pune 411 007, India

**Additional File 1: Figure S1**

**
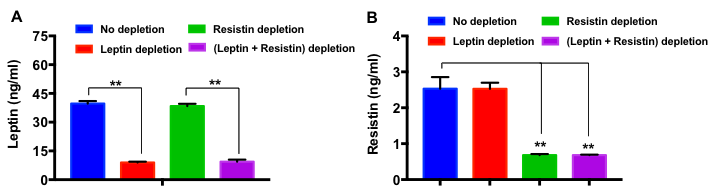
**

**Figure S1: Validation of immunodepletion of leptin form serum collected from HFD C57BL/6J mice.** Serum from HFD C57BL/6J mice was collected, and pooled. Leptin and resistin (or both together) were immunodepleted from the serum by incubating it with respective specific antibody at 4°C for overnight. Antigen-antibody complexes were precipitated using protein A/G-plus agarose beads by incubating at 4°C for 4 h. Next, the supernatant containing immunodepleted serum was collected by centrifuging the tubes at 10,000 rpm at 4°C. Immunodepletion of leptin (**A**) and resistin (**B**) in serum was confirmed by ELISA in triplicates. The results are given as means ± standard deviation. Statistical analysis was performed using two-tailed unpaired Student’s t test; **, *p* < 0.001.

**Additional File 1: Figure S2**

**
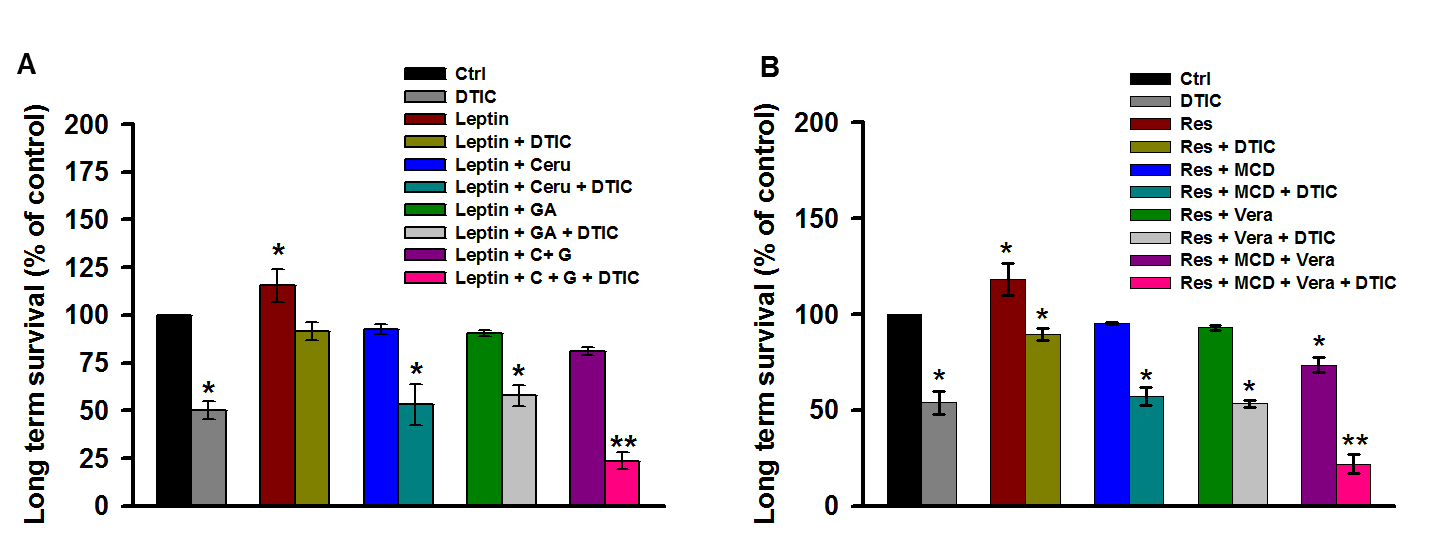
**

**Figure S2:** A375 cells were cultured in the presence of leptin or resistin along with inhibitors for 48 h. Next, the medium was changed and fresh medium was added. (**A**) Representative image showing the long term survival of A375 cells in the presence or absence of leptin together with inhibitors. (**B**) Representative image showing the long term survival of A375 cells in the presence or absence of resistin together with inhibitors. The data were quantified using Image J software. The results are given as means ± standard error of the mean. All the experiments were performed three times. Statistical analysis was performed using two-tailed unpaired Student’s t test; *, *p* < 0.05, **, *p* < 0.001; Ctrl- Control, Res- Resistin, Chx- Cycloheximide, Ceru or C- cerulenin; GA or G- Geldanamycin.

**Additional File 1: Table S1**

**
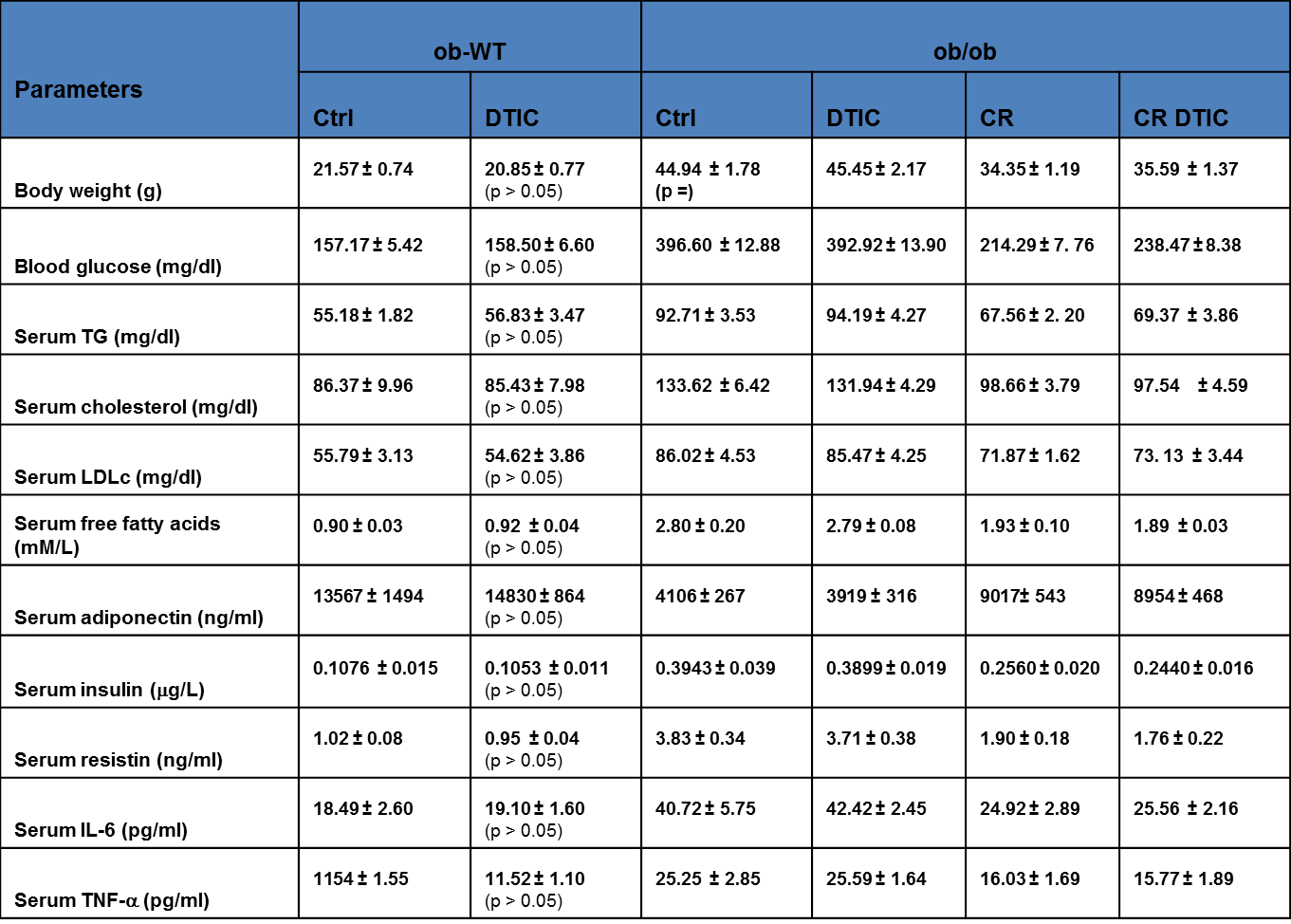
**

**Table S1: Evaluation of obesity-associated factors in WT and db/db mice.** ob/ob mice were divided into two major groups. One group was fed *ad libitum* on normal diet. In the second group, caloric intake was restricted to 50% by providing half the quantity of feed before inoculating B16F10 cells. After 15 days, mice of all groups were injected subcutaneously with B16F10 cells (2 × 10^5^ cells/mouse in 100 µl PBS). After tumor formation, vehicle or DTIC treatment (N = 6 per each group) was given as per the experimental layout shown in Figure 4. Parallely, similar experiment was performed in ob-WT mice (N = 6 per each group). Body weight of all the mice was monitored weekly throughout the study, and serum was collected at the end of the experiment.

**Additional File 1: Table S2**

**
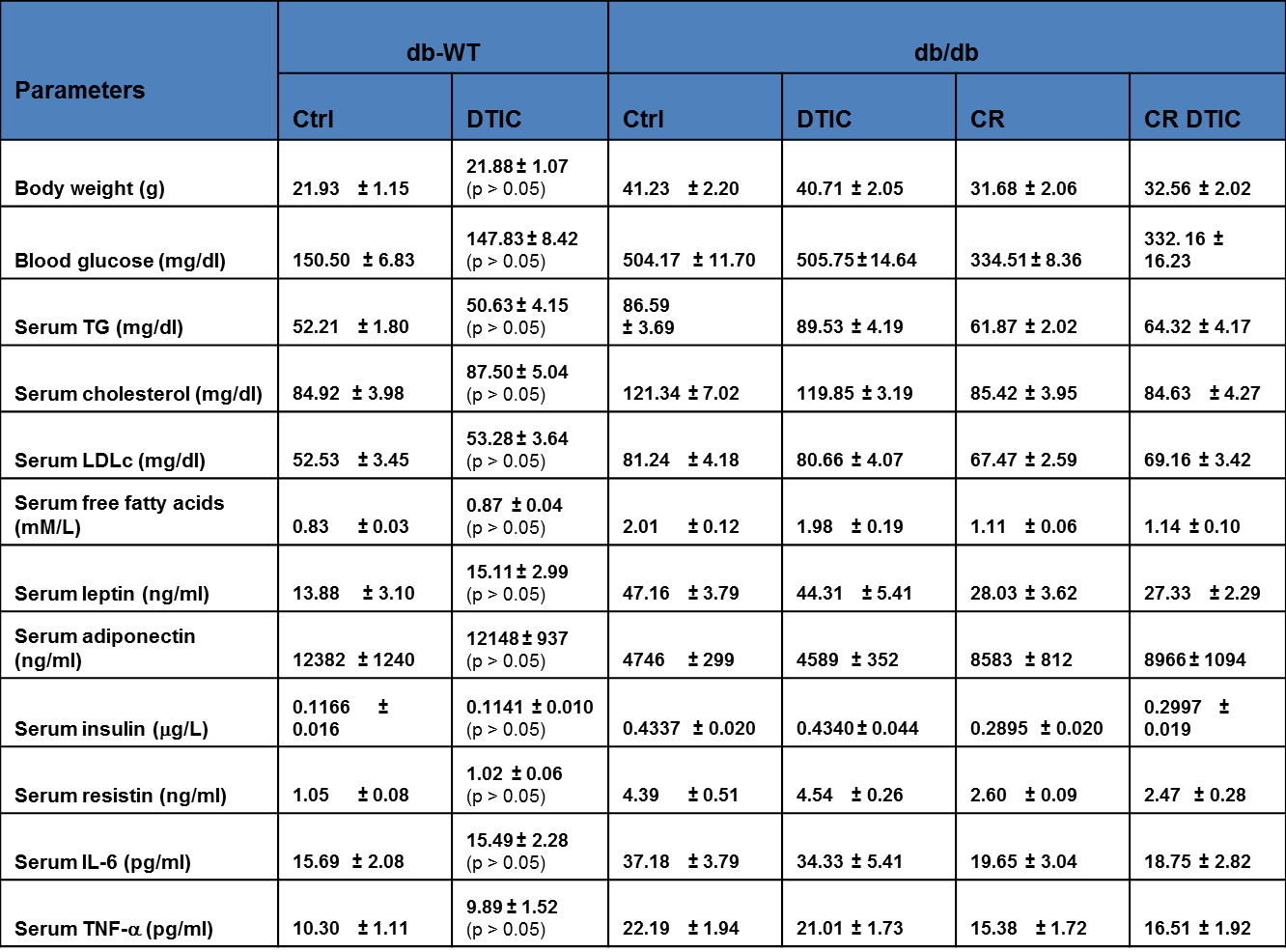
**

**Table S2: Evaluation of obesity-associated factors in WT and db/db mice.** db/db mice were divided into two major groups. One group was fed *ad libitum* on normal diet. In the second group, caloric intake was restricted to 50% by providing half the quantity of feed before inoculating B16F10 cells. After 15 days, mice of all groups were injected subcutaneously with B16F10 cells (2 × 10^5^ cells/mouse in 100 µl PBS). After tumor formation, vehicle or DTIC treatment (N = 6 per each group) was given as per the experimental layout shown in Figure 4. Parallely, the similar experiment was performed in db-WT mice (N = 6 per each group). Body weight of all the mice was monitored weekly throughout the study, and serum was collected at the end of the experiment.
